# Procedural Comparison of the Amulet Versus Watchman Devices: A Single Center’s 'Change of Heart'

**DOI:** 10.7759/cureus.103404

**Published:** 2026-02-11

**Authors:** Marc T Zughaib, Andrew Sherman, Wadie David, Keyur Patel, Brandon Grodman, Marcel E Zughaib, Christopher Bradley

**Affiliations:** 1 Cardiology, Henry Ford Providence Southfield Hospital, Southfield, USA; 2 Internal Medicine, Henry Ford Providence Southfield Hospital, Southfield, USA

**Keywords:** amulet, interventional and structural cardiology, left atrial appendage, left atrial appendage occluder, left atrial appendage occlusion device, watchman

## Abstract

Background

The recommendation level for left atrial appendage occlusion (LAAO) via device implantation has increased in the most recent American College of Cardiology (ACC) guidelines, resulting in an increase in implantation rates. Our center had the unique experience of originally implanting Watchman device^TM ^(Boston Scientific, Marlborough, MA, US) devices from 2017 to 2021 and abruptly switching to implanting Amplatzer Amulet^TM ^(Abbott Cardiovascular, Plymouth, MN, USA) devices from 2021 to the current day. This sudden switch between devices created a unique opportunity for direct comparisons of the two LAAO devices. Our study aimed to compare the differences between these two devices regarding procedural time, radiation dose, and contrast use.

Methods

Several patients (n=1262) underwent LAAO implantations from 2017-2024 at our center. We performed simple random sampling to select 200 patients that received the Watchman devices and 200 patients that received the Amulet devices. Demographic information, procedural time, radiation dose, and contrast doses were collected for all of them. Student’s t-tests were performed on continuous variables for statistical analyses.

Results

The average procedure time overall was 56.71 minutes. The average radiation dose of patients undergoing the Watchman device implantation was 167.5 mGy (95% CI: 140.9-194.1) and 247.0 mGy (95% CI: 201.4-286.7) for the Amulet device (p=0.001). The average procedure time did not differ as it was 52.4 minutes (95% CI: 48.7-56.1) for the Watchman device implantation versus 56.1 minutes for the Amulet implants (95% CI: 52.1-60.1; p=0.183). The average contrast dose also did not differ, as patients undergoing the Watchman device implantation used 31.3 cc (95% CI: 27.8-34.8), whereas it was 34.5 cc (95% CI: 31.4-37.7; p=0.176) with the Amulet device.

Conclusion

There was a significantly higher radiation dose associated with the implantation of the Amulet versus the Watchman devices. There were no significant differences between procedural time or contrast use between the two devices.

## Introduction

Atrial fibrillation (AF) represents an evolving public health concern in the United States due to its association with increased mortality and thromboembolic events. The incidence is projected to increase from 1.2 million to 2.6 million cases between 2010 and 2030 [1,2]. Validated risk scores (Congestive heart failure, Hypertension, Age ≥75 years (doubled), Diabetes mellitus, prior Stroke or Transient Ischemic Attack (TIA) or thromboembolism (doubled), Vascular disease, Age 65 to 74 years, Sex category (CHA_2_DS_2_-VASc); Anticoagulation and Thromboembolism Risk In Atrial Fibrillation (ATRIA); and Global Anticoagulant Registry in the FIELD-Atrial Fibrillation (GARFIELD-AF)) guide decisions regarding anticoagulation [3-6]. However, the use of anticoagulation is often challenging due to the risk of serious bleeding, and additional risk scores like Hypertension, Abnormal Renal/Liver Function, Stroke, Bleeding History or Predisposition, Labile INR, Elderly, Drugs/Alcohol Concomitantly (HAS-BLED) and ATRIA, are utilized to further evaluate risks versus benefits before initiating, or continuing therapy [5-8]. 

The left atrial appendage (LAA) accounts for up to 90% of thrombi in non-valvular AF [2,8]. This predisposition to thrombus formation is attributed to the LAA’s unique anatomical and physiological features, which, in the context of AF, promote blood stasis [2]. Therefore, the LAA serves as a prime target for emerging stroke prevention strategies, particularly for high-risk patients who are intolerant of long-term anticoagulation. 

Left atrial appendage occlusion (LAAO) has emerged as a significant alternative to anticoagulation for stroke prevention in AF. The two most frequently utilized occluder devices include Amplatzer Amulet^TM ^(Abbott Cardiovascular, Plymouth, MN) and Watchman^TM ^device (Boston Scientific, Marlborough, MA) [9]. Both devices have demonstrated individual efficacy and feasibility [10,11]. Head-to-head comparisons between procedural variables of the two devices have not been tested in clinical trials and operators continue to have their own individual biases and preferences for their preferred device. 

Beyond the primary clinical endpoints of stroke prevention, procedural complications, mortality, procedural efficiency, and safety are crucial factors to consider in LAAO implantation. Understanding and comparing these procedural metrics between Watchman and Amulet can provide insights into the practical aspects of device implantation. Our center had a unique opportunity as a high volume LAAO device implantation site that switched contracts from Boston Scientific to Abbott Cardiovascular. This led to our study designed to investigate differences in procedural variables including time of procedure, contrast use, and radiation exposure. 

## Materials and methods

There were a total of 1,262 patients who underwent LAAO implantations from 2017 to 2024 at our institution (Henry Ford Providence Southfield Hospital, Southfield, USA). We performed simple random sampling to select 200 patients who received Watchman devices and 200 patients who received Amulet devices. These patients were selected as our institution’s contract with LAAO device companies changed from Boston Scientific to Abbott Cardiovascular in 2021, which led to a switch from using one device to the other. Random sampling was performed to minimize the effect of an operator learning curve. 

Patients were included if they had nonvalvular atrial fibrillation with a CHA_2_DS_2_-VASc score ≥2 and an appropriate clinical rationale to seek a nonpharmacologic alternative to long-term oral anticoagulation. Inclusion criteria consisted of patients older than 18 years who were undergoing LAAO implantation. All of these patients must have met specific guideline criteria to qualify for LAAO implantation. Exclusion criteria included incomplete documentation of variables, unretrievable data, technical/anatomical variants, or aborted cases due to LAA thrombus noted during pre-procedural transesophageal echocardiogram (TEE). 

Data collected included demographic information, CHA_2_DS_2_-VASc scores, HAS-BLED scores, procedural time, radiation dose, contrast doses, new pericardial effusions, and pericardial effusions that required emergent pericardiocentesis. Radiation dose and contrast dose were populated into the procedure reports automatically. Continuous variables were presented as means with 95% confidence intervals. Categorical variables were presented as frequencies and percentages. Comparisons between the Amulet and Watchman device groups were performed using independent samples t-tests for continuous variables and chi-square tests or Fisher's exact tests for categorical variables, as appropriate. The study was reviewed and approved by the Expedited Institutional Review Board Committee of the Henry Ford Health (approval number 18554). 

## Results

Between 2017 and 2024, a total of 1,262 patients underwent LAA occlusion at our institution. After simple random sampling to minimize operator learning-curve effects, 400 patients were included in this analysis: 200 who received the Watchman device and 200 who received the Amulet occluder. The institutional transition from Boston Scientific to Abbott Cardiovascular devices occurred in 2019. 

In the overall cohort, the average age was 76.36 years (95% Confidence Interval: 75.5-77.21) with an average BMI of 29.99 (95% CI: 28.99-30.98) (Table 1). 

**Table 1:** Overall age, gender, BMI, CHA₂DS₂-VASc, and HAS-BLED scores of patients included in the study (n=400) The average age, gender, BMI, CHA₂DS₂-VASc, and HAS-BLED scores of patients that received Amulet (n=200) and Watchman (n=200) devices are included with p-values. CHA2DS2-VASc: Congestive heart failure, Hypertension, Age ≥75 years (doubled), Diabetes mellitus, prior Stroke or Transient Ischemic Attack (TIA) or thromboembolism (doubled), Vascular disease, Age 65 to 74 years, Sex category; HAS-BLED:  Hypertension, Abnormal Renal/Liver Function, Stroke, Bleeding History or Predisposition, Labile INR, Elderly, Drugs/Alcohol Concomitantly.

Characteristic	Overall population (Mean)	Amulet group (Mean, 95% CI)	Watchman group (Mean, 95% CI)	p value (Amulet vs Watchman)
Age (years)	76.4	76.67 (75.5–77.8)	76.04 (74.8–77.3)	0.475
Gender (Male, %)	222 (56%)	121 (61%)	101 (51%)	0.18
BMI (kg/m²)	29.99	30.6 (28.8–32.4)	29.4 (28.5–30.2)	0.20
CHA₂DS₂-VASc score	4.5	4.43 (4.27–4.59)	4.57 (4.38–4.76)	0.25
HAS-BLED score	3.05	3.04 (2.94–3.14)	3.06 (2.92–3.21)	0.835

The average CHA_2_DS_2_-VASc score was 4.5 (95% CI: 4.34-4.58), and the HAS-BLED score was 3.05 (95% CI: 2.98-3.13). The overall radiation dose was 207.16 mGy (95% CI: 183.02-231.3), procedure time of 54.3 minutes (95% CI: 51.53-57.0), and contrast dose was 32.9 cc (95% CI: 30.55-35.26) (Table 2). 

**Table 2:** Overall study population (n=400) procedure time, radiation dose, and contrast dose Average procedure time, radiation dose, and contrast dose for patients who received the Amulet (n=200) and Watchman (n=200) devices are included with p-values.

	Study population	Amulet group	Watchman group	p-value
Procedure time (min)	54.3	56.1	52.4	0.183
Radiation dose (mGy)	207.2	247.0	167.5	0.001
Contrast dose (cc)	32.9	34.5	31.3	0.176

**Figure 1:**
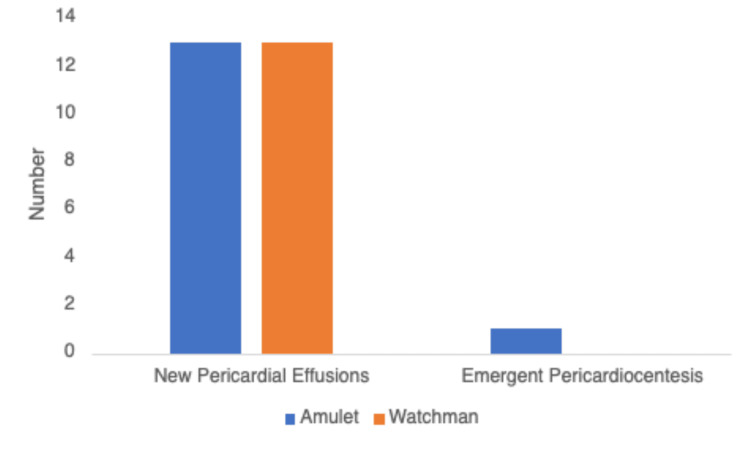
Number of new pericardial effusions and those that required emergent pericardiocentesis There were 13 new pericardial effusions noted in each group, with one in the Amulet group requiring emergent pericardiocentesis.

The total number of post-deployment pericardial effusions were 26. The total number of post-deployment pericardiocentesis required was one (Figure 1). 

The average age, BMI, CHA_2_DS_2_-VASc score, and HAS-BLED scores were similar between the two patient populations (Table 1). 

The average radiation dose (Air Kerma) of patients undergoing Watchman device implantation was 167.5 mGy (95% CI: 140.9-194.1), which increased in those undergoing Amulet device implantation to 247.0 mGy (95% CI: 201.4-286.7; p=0.001) (Table 2). 

The average procedure did not differ as the Watchman device implantation was 52.4 minutes (95% CI: 48.7-56.1), versus 56.1 minutes in Amulet implants (95% CI: 52.1-60.1; p=0.183). The average contrast dose did not differ, as patients undergoing implantation with the Watchman device used 31.3 cc (95% CI: 27.8-34.8), whereas those with the Amulet device used 34.5 cc (95% CI: 31.4-37.7; p=0.176). 

The total number of pericardial effusions observed in patients undergoing Watchman implantation was 13 (6.5%), with no patients requiring pericardiocentesis (Figure 1). The total number of pericardial effusions observed in patients who underwent Amulet implantation was 13 (6.5%), with one patient requiring emergent pericardiocentesis. 

## Discussion

This single-center study provides insight into the procedural elements of percutaneous LAAO with the Amulet and Watchman devices. Our center was uniquely positioned to directly study these devices, as Watchman devices were exclusively used from 2017-2021. Then, Amulet devices were exclusively available from 2021 to the present day. Overall, there were no significant differences between the demographics, CHA_2_DS_2_-VASc scores, HAS-BLED scores, procedure time, or contrast dose in patients receiving either device in our study. However, there was a significantly higher level of radiation associated with the Amulet device procedure when compared to the Watchman device. 

Concerns regarding higher radiation exposure associated with the Amulet relative to the Watchman device extend beyond immediate procedural risks. Exposure of both patients and medical staff to ionizing radiation during fluoroscopy has been a concern with regard to short- and long-term safety [12,13]. Increased fluoroscopy time between the two procedures would be related to several procedural factors. The Amulet device has two components (Disc and Lobe). Optimal positioning of each component may add procedural complexity. The Watchman sheath has integrated components, whereas the Amulet sheath has required connections between the device loader and delivery sheath, which includes a hemostatic valve and a sheath adapter for connecting a 14F sheath to devices sized 16-25 mm [14]. 

The criteria to deploy the devices are different and may contribute to the difference in fluoroscopic time. The Watchman device requires confirmation of deployment by compression, adequate seal, and stability of a single component, visualized via TEE/ Intracardiac Echocardiography (ICE). The Amulet device is complicated by the presence of a disc and lobe. The body of the lobe should be two-thirds past the circumflex artery, with some compression within the LAA, and oriented perpendicular to the long access of the LAA. Next, the sheath is retracted to expose the disc and create separation between the lobe. The disc should not excessively overlay the coumadin ridge of the pulmonary vein and mitral valve, respectively. Once this device is deployed, fluoroscopy is used to confirm the appropriate stability position and proximity to vascular structures such as the pulmonary artery. Subsequently, TEE/ICE are used to confirm the position [14]. 

Despite the additional fluoroscopy needed to confirm the Amulet device's placement, there were no significant differences between the length of the procedure or the average contrast dose usage. A potential explanation for this finding was the operator's prior experience with LAAO. Contrast use was similar between the two groups as there is typically limited contrast required for either procedure. Small injections of contrast are used to visualize the anatomy and any potential peri-device leak [14].

Prior studies have not specifically investigated procedural variables in a single center that has performed high volumes of implantations with both devices. There have been prior studies investigating the safety and efficacy of first and second generations of LAAO devices designed by Amplatzer, specifically comparing the Cardiac Plug (first generation) and Amulet (second generation) [15]. The Amulet device was noted to have a significant reduction in fluoroscopy time, radiation dose, and contrast dye used, when compared to the Cardiac Plug. These findings were attributed to a simpler implantation procedure as well as improved design [15]. 

There were 26 total new pericardial effusions in our study, with equal distribution between the patients that received the Amulet device or the Watchman device. However, one patient in the Amulet group required emergent pericardiocentesis. Device-related trauma remains the prevalent theory regarding the incidence of pericardial effusions. The Amulet device has prongs that anchor the device within the LAA, which can also increase the chance of localized trauma. This mechanism has been theorized for the reason this patient experienced a pericardial effusion post-deployment. While not statistically significant in our study, prior studies have noted similar rates of pericardial effusions, ranging from 0.5-5% [16-18]. A limitation of the study is the retrospective nature, and adjudication of effusions were limited. 

The limitations to our study include a single-center experience, with a relatively low number of patients included in the study. The operators included in the study all perform high volume LAAO procedures, which provided a high level of expertise in deploying the devices. However, the registry used does not have complete data in terms of long term follow-up, i.e., six months after the implantation of the devices. This is due to a diasporic referral pattern of private practices, within and outside the implanting institution, some with alternate electronic medical records. Future studies with longer follow-up are needed to determine whether modest differences in procedural variables translate into clinically meaningful differences in patient outcomes. 

## Conclusions

We presented a unique single-center experience, comparing procedural variables during the Amulet versus Watchman device implantation. There were significantly higher levels of radiation exposure during the Amulet procedures. However, the length of the procedure, contrast dose exposure, number of pericardial effusions, and emergent pericardiocentesis were not significantly different. This study highlights a unique intraprocedural analysis of these increasingly popular devices. Further studies are recommended to validate these findings. 
